# Suppression of low-energy dissociative electron attachment in Fe(CO)_5_ upon clustering

**DOI:** 10.3762/bjnano.8.219

**Published:** 2017-10-20

**Authors:** Jozef Lengyel, Peter Papp, Štefan Matejčík, Jaroslav Kočišek, Michal Fárník, Juraj Fedor

**Affiliations:** 1 J. Heyrovský Institute of Physical Chemistry v.v.i., Czech Academy of Sciences, Dolejškova 3, 18223 Prague 8, Czech Republic; 2 Institut für Ionenphysik und Angewandte Physik, Leopold-Franzens-Universität Innsbruck, Technikerstrasse 25, 6020 Innsbruck, Austria; 3 Department of Experimental Physics, Faculty of Mathematics, Physics and Informatics, Comenius University, Mlynská dolina F2, 84215 Bratislava, Slovakia

**Keywords:** aggregation effects, dissociative electron attachment, FEBID, iron pentacarbonyl, long-range interactions

## Abstract

In this work, we probe anion production upon electron interaction with Fe(CO)_5_ clusters using two complementary cluster-beam setups. We have identified two mechanisms that lead to synthesis of complex anions with mixed Fe/CO composition. These two mechanisms are operative in distinct electron energy ranges. It is shown that the elementary decomposition mechanism that has received perhaps the most attention in recent years (i.e., dissociative electron attachment at energies close to 0 eV) becomes suppressed upon increasing aggregation of iron pentacarbonyl. We attribute this suppression to the electrostatic shielding of a long-range interaction that strongly enhances the dissociative electron attachment in isolated Fe(CO)_5_.

## Introduction

In recent years a number of gas-phase studies on molecules that are commonly used as precursors in electron-induced nanofabrication have stressed the importance of electrons with energy below 1 eV [1–3]. In many cases, these cause the cleavage of one metal–ligand bond via dissociative electron attachment (DEA) and corresponding cross sections reach unusually high values [2–3]. Iron pentacarbonyl, Fe(CO)_5_, is no exception: the dominant DEA product is Fe(CO)_4_^−^ [4] and high thermal electron electron attachment rates measured in flowing afterglow (8 × 10^−8^ cm^3^ s^−1^) suggest high DEA cross section at very low electron energies [5]. The dissociative processes at such low electron energies (up to a few hundreds of meV) are very sensitive to the immediate environment of the active molecule [6–8].

Several effects can influence the DEA outcome. For example, the DEA in this energy range is strongly enhanced in many molecules by long-range interactions (e.g., electron-induced dipole) [7], which can be effectively electrostatically shielded by the environment [9]. Another common effect is that the formation of the transient anion polarizes the environment and the additional polarization energy shifts the energy of the repulsive anion potential curve with respect to the curve of the neutral molecule [10]. A small shift then results in a large change (increase) in the DEA cross section, since this is extremely sensitive to the overlap of the two curves around the Franck–Condon region [11]. Finally, the target molecule can be stabilized by mechanical suppression of the dissociation (caging) and energy transfer to the environment [12–13]. The environment can thus both enhance and suppress the low-energy DEA reactions and it is difficult to assess its effect a priori.

Clusters represent an ideal tool for studying the effect of an environment since they allow for using the same experimental approach as the gas phase studies (crossed electron – target beam with product mass analysis) and enable direct comparisons for various aggregation degrees (gas phase – small clusters – large clusters). When compared to the surface-based ion desorption approach, cluster-beam experiments do not suffer from desorption probability problem. Once a fragment ion is created, it can be detected via mass spectrometry, regardless of whether it separated from the rest of the cluster (”desorption”) or stayed attached to the rest of the cluster (”no desorption”).

Our recent experiments [14] on precursor molecules adsorbed on large argon nanoparticles showed that different energy ranges start to play a role in electron attachment: the mixed metal/ligand species are produced by a self-scavenging mechanism. This is a process where an electron with intermediate electron energies (5 to 20 eV) electronically excites one precursor molecule that undergoes neutral dissociation. This yields an electron with very low residual energy that causes DEA in another precursor molecule and the resulting anion effectively reacts with the coordinatively unsaturated products of the neutral dissociation. Such a process – possibly very relevant at realistic FEBID conditions – is operative in higher electron energy ranges and represents a synthesis mechanism in the deposits.

In this paper, we provide detailed insight into both relevant energy ranges (near-zero eV and intermediate 5–20 eV) and changes in the dissociative electron attachment behavior with varying aggregation stage. We probe anion production in two distinct types of clusters: (i) pure small Fe(CO)_5_ clusters and (ii) small Fe(CO)_5_ aggregates deposited on large argon nanoparticles (Ar*_N_*, 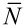 ≈ 200). The presence of the environment leads to a complete suppression of the DEA signal close to 0 eV. We ascribe this effect primarily to shielding of the long-range electron–molecule interactions due to the polarizable nature of the environment.

## Experimental

Two experimental setups have been used, both probing the negative ion production in various Fe(CO)_5_ clusters.

The first is a cluster beam (CLUB) apparatus located in Prague, Czech Republic [12,15]. The clusters were produced by a supersonic expansion into vacuum, and the beam was skimmed and passed through three differentially pumped chambers (one containing a pickup cell) into an interaction chamber where it was crossed with an electron beam of variable energy. Two different modes of operation that produce distinctly different types of clusters were utilized. In the first one, a mixture of Fe(CO)_5_ with argon at a stagnation pressure of 1 bar was expanded though a conical nozzle with 55 μm diameter. The nature of the resulting clusters was probed by recording positive ion mass spectra. Only cluster ions of the mixed Fe/CO composition were detected. Even though this does not fully exclude the possibility that the neutral Fe(CO)_5_ clusters have several Ar atoms attached to them (that evaporate during ionization [16]), we conclude that in the first mode, the dominant species in the beam are pure Fe(CO)_5_ clusters. In the second mode, described in detail in our previous publications [13–14], the pure argon gas was expanded under a stagnation pressure of 5 bar through a conical nozzle of 50 μm diameter at a temperature *T*_0_ = 223 K, which leads to the production of pure argon nanoparticles with a mean size of 200 atoms. The mean size was determined by using empirical scaling formulas as described in our earlier works [15,17]. Iron pentacarbonyl vapor was then introduced into the pickup cell and by using sufficiently high local pressures, multiple guest Fe(CO)_5_ molecules were picked-up. They coagulate efficiently and form small Fe(CO)_5_ aggregates on the surface of the argon nanoparticles.

The electron beam in CLUB was produced in a three-lens focusing electron gun. The created anions were then analyzed in a reflectron time-of-flight mass spectrometer (RTOF). The whole experiment was pulsed: the electron beam passed the interaction region for 1 μs while it was field free and then the extraction field of 2 kV/cm was applied to accelerate anions into the RTOF. The repetition frequency was 10 kHz. The electron-energy scale has been calibrated using the 2.2 eV resonance in the O^−^ production from N_2_O. The electron gun has been constructed primarily for producing high current at higher electron energies (70 to 100 eV, typical in positive ion mass spectroscopy), it has thus two disadvantages: the electron-beam resolution is around 600 meV and below 1.5 eV the electron current drops to low values and is difficult to control.

We have performed analogous experiments on the cluster-beam (CLUSTER) setup located in Bratislava, Slovakia, originally built at the Free University in Berlin. Only the first type of target (pure Fe(CO)_5_ clusters) could be probed on CLUSTER. The beam was formed by supersonic expansion of 1:250 of Fe(CO)_5_/Ar via a 75 μm nozzle (stagnation pressure 2–3 bar), skimmed and passed to a differentially pumped reaction chamber. There it collided with an electron beam formed perpendicularly to the cluster with a trochoidal electron monochromator. The electron energy was calibrated to the 0 eV resonance of electron attachment to SF_6_ molecule. Contrary to the CLUB setup, with the electron monochromator in the CLUSTER experiment, we are able to tune the electron energy resolution below 100 meV. However, due to low DEA signals we operated the monochromator at the electron energy resolution of 300 meV, as determined from the full width at half maximum of the SF_6_^−^ peak. The anions produced were separated in a quadrupole mass analyzer according to their mass charge ratios *m*/*z*.

The two cluster-beam setups used in the present work are complementary. The one in Prague (CLUB) has higher sensitivity due to higher electron currents (non-monochromatized beam) and the time of flight mass analyzer exhibits higher mass resolution than the quadrupole system. The TOF system also suffers from much less discrimination towards various masses and it thus provides more reliable fragment mass distributions. The CLUSTER setup in Bratislava, on the other hand, has an advantage of much better electron-energy resolution and performance at low electron energies. Their combination represents a powerful technique with which both low signals, isotope distributions and high-energy resolution ion yields can be obtained.

## Results

**Figure 1:**
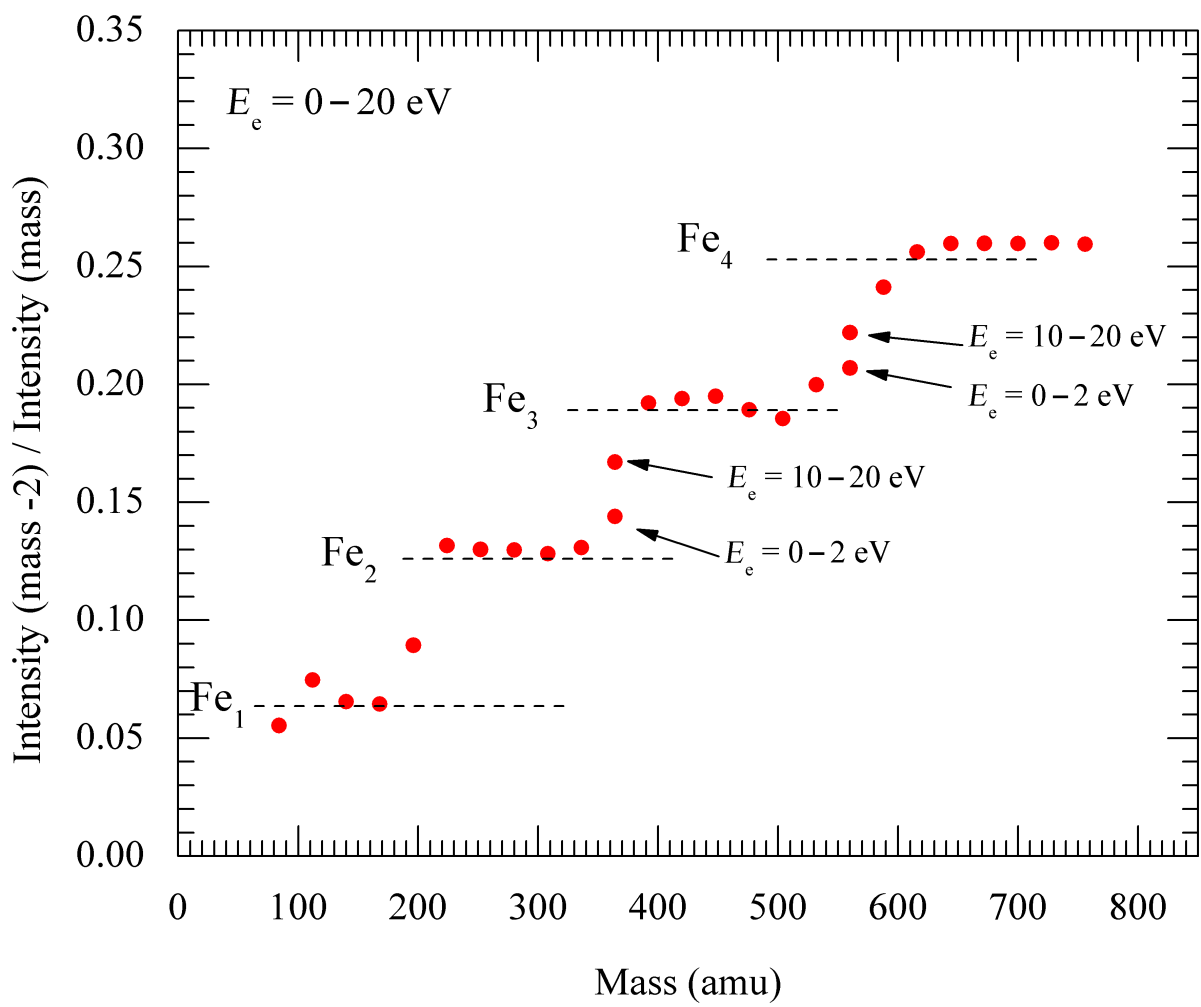
Intensity ratio of the anion mass peak with ^54^Fe isotope to the peak with ^56^Fe isotope, reflecting the number of iron atoms in the anion. Measurements were performed on the CLUB setup.

The mass spectrometry analysis of anions resulting from electron interactions with Fe(CO)_5_ clusters is complicated by the fact that two CO ligands have the same mass as one iron atom (56 amu). We have used the fact that the iron atom has an isotope at 54 amu (6.3% abundance) which CO is lacking, where the oxygen and carbon have isotopes with mass higher than the main one. For each detected anion fragment, the inverse ratio of the main peak (multiple of 28) to the peak two masses lower (i.e., the ratio intensity(mass-2)/intensity(mass)) reflects the number of iron atoms in the anion (12.6% for two Fe atoms, 18.9% for three Fe atoms). Due to the low abundances of the weak isotopes, such an analysis was possible only on the more sensitive CLUB setup – Figure 1 shows the obtained ratios for electron attachent to pure Fe(CO)_5_ clusters. The same analysis for Fe(CO)_5_ aggregates deposited on argon has been published [14] and shows very similar results. Almost for all mass peaks, the experimental ratios clearly reveal the number of iron atoms and thus chemical composition of the anions. The analysis has been done from the cumulative mass spectra (sum of all the mass spectra at electron energies in the range 0–20 eV), however, for peaks at masses 364 and 560 amu the isotope ratio turned out to be electron-energy dependent. At low electron energies, the ions Fe_2_(CO)_9_^−^ and Fe_3_(CO)_14_^−^, respectively, contribute dominantly to these mass peaks. At higher electron energies, these peaks correspond mostly to Fe_3_(CO)_7_^−^ and Fe_4_(CO)_12_^−^.

**Figure 2:**
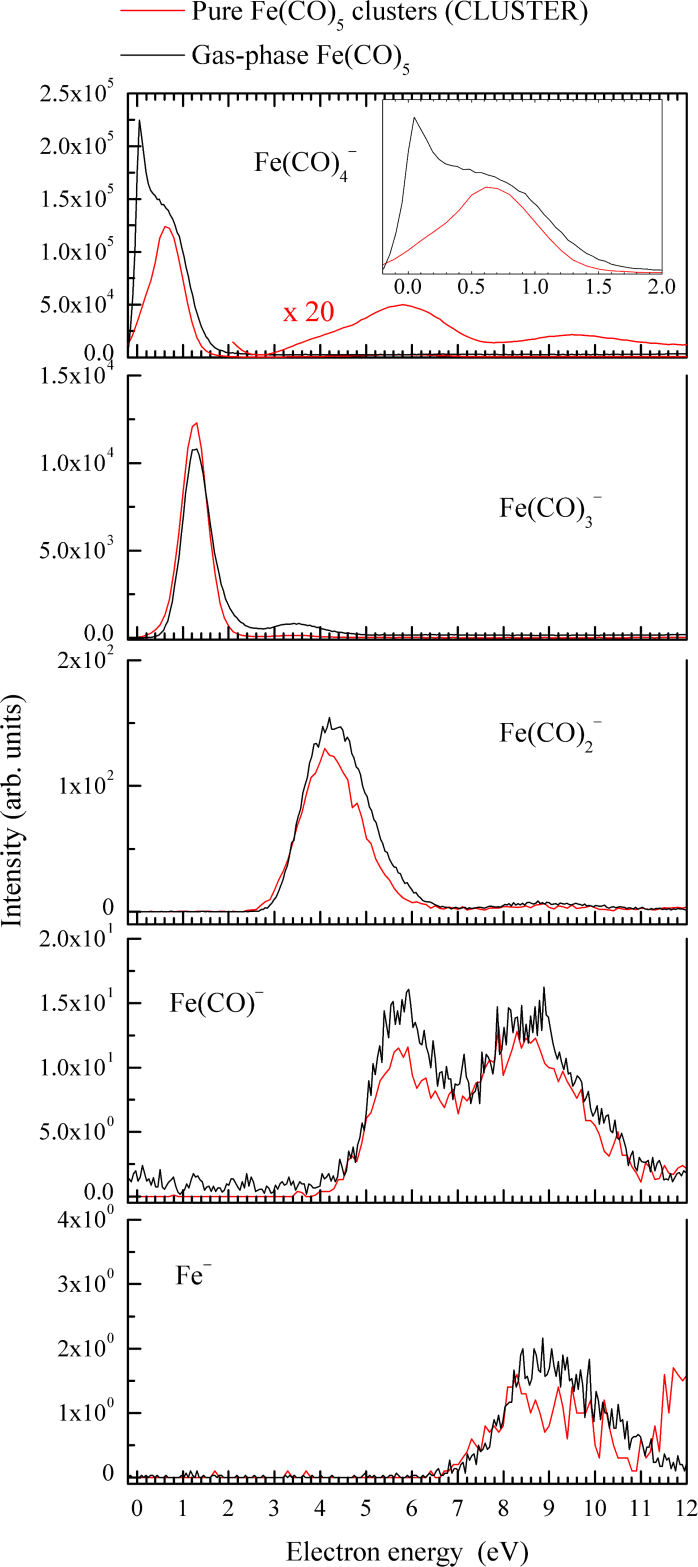
Red lines: Yield of anions containing one iron atom, following DEA to pure Fe(CO)_5_ clusters as measured using the CLUSTER setup. Black lines: Anion yields from the gas-phase Fe(CO)_5_ [18].

Figure 2 shows the yields of monomeric anions (those containing one iron atom) from pure Fe(CO)_5_ clusters. The data were taken on the CLUSTER setup and are compared with the anion yields from gas-phase iron pentacarbonyl [18], measured in effusive molecular beam with a similar trochoidal electron monochromator. The most striking difference is in the strongest fragment Fe(CO)_4_^−^: the sharp peak close to 0 eV disappears and the maximum shifts to 0.65 eV. This is not an instrumental effect of the CLUSTER setup (lack of low-energy electrons in the incident beam). The SF_6_^−^ signal from SF_6_ peaks at electron energies 0.65 eV lower than Fe(CO)_4_^−^ from iron pentacarbonyl clusters.

**Figure 3:**
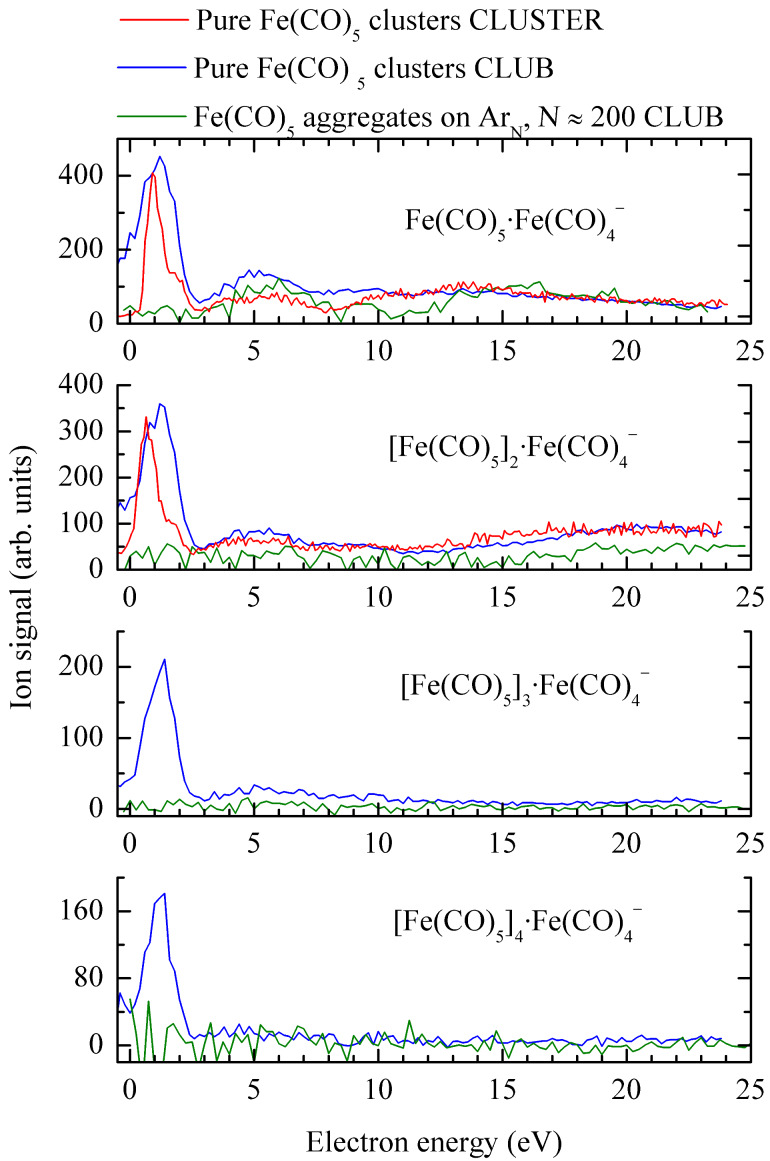
Anion yield of the form [Fe(CO)_5_]*_m_*·Fe(CO)_4_^−^. Red: high-resolution data for pure Fe(CO)_5_ clusters from the CLUSTER setup (Bratislava), blue: high-sensitivity data for pure Fe(CO)_5_ clusters from the CLUB setup (Prague), green: high-sensitivity data for Fe(CO)_5_ aggregates deposited on Ar*_N_* nanoparticles, 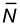 ≈ 200. The intensity corresponds to CLUB measurements (high transmission and low discrimination of the RTOF analyzer), the other curves were arbitrarily scaled.

Figure 3 shows the yields of anions where the dominant fragment Fe(CO)_4_^−^ is bound to intact monomeric units [Fe(CO)_5_]*_m_*·Fe(CO)_4_^−^, *m* = 1….4. The high resolution CLUSTER data for pure Fe(CO)_5_ clusters show that the low-energy peaks have maxima around 0.65 eV, similar as Fe(CO)_4_^−^ in Figure 2. The lower resolution CLUB data agree very well and provide the ion yields also for very weak fragments. Finally, we show the CLUB data for aggregates deposited on argon nanoparticles. Here, the DEA signal in these channels is suppressed below the detection limit in the whole low-energy range (below 2 eV). Unfortunately, it is not possible to verify this effect also for the production of Fe(CO)_4_^−^, since in the pickup experiment, a certain amount of gas-phase iron pentacarbonyl diffuses into the interaction region and causes a strong Fe(CO)_4_^−^ background signal. In any case, this signal demonstrates that the disappearance of the low-energy anion signal in the pickup experiment is not due to the absence of slow electrons in the electron beam and has to have a real physical origin.

**Figure 4:**
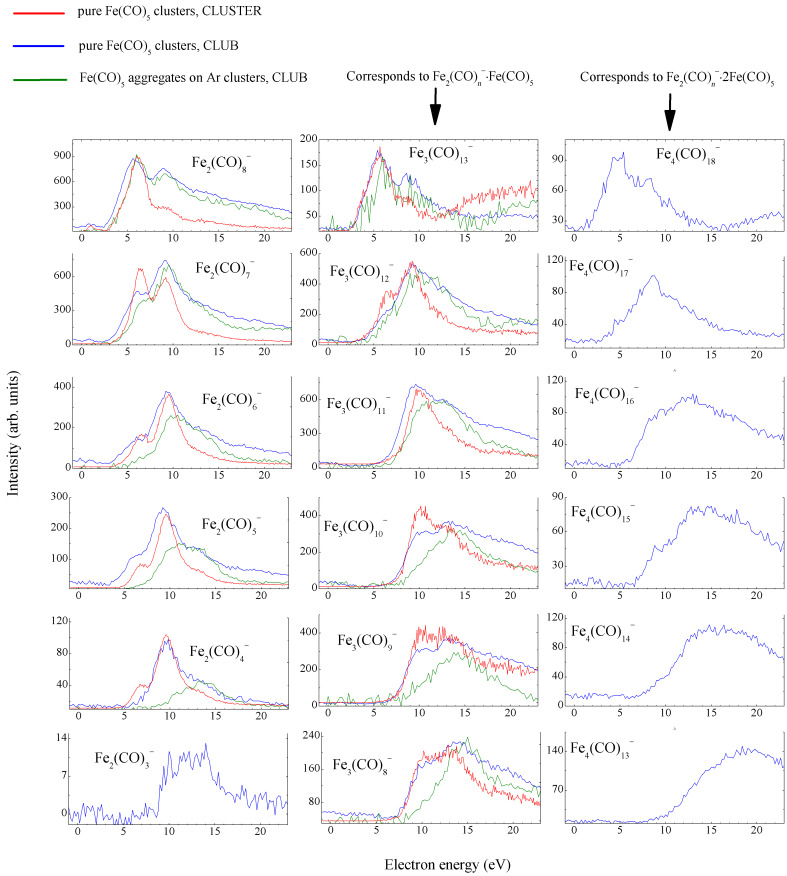
Additional anion yields. Red: high-resolution data for pure Fe(CO)_5_ clusters from the CLUSTER setup (Bratislava), blue: high-sensitivity data for pure Fe(CO)_5_ clusters from the CLUB setup (Prague), green: high-sensitivity data for Fe(CO)_5_ aggregates deposited on Ar*_N_* nanoparticles, 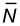 ≈ 200. The intensity corresponds to CLUB measurements (high transmission and low discrimination of the RTOF analyzer), the other curves were arbitrarily scaled.

Figure 4 shows the energy-dependent yields of fragments anions other than [Fe(CO)_5_]*_m_*·Fe(CO)_4_^−^. In contrary to those, anions in Figure 4 do not show any signal at low electron energies and are produced only above 5 eV. The panels are ordered such, that there is always one monomer Fe(CO)_5_ unit separating the neighbouring columns. The similarity of spectra in individual rows leads us to the conclusion that the structure of anions containing more than two iron atoms corresponds to a ”core” anion containing two iron atoms with one or two intact monomer units attached to it. The CLUB data for pure Fe(CO)_5_ clusters and for Fe(CO)_5_ aggregates adsorbed on large Ar nanoparticles are very similar, with one notable difference: with the decreasing number of ligands in the fragment anion, the band between 5 and 10 eV disappears when the aggregates are adsorbed on argon. At this place we note, that in the whole energy range, the electron attachment to argon-adsorbed Fe(CO)^+^ does not lead to production of any mixed argon–iron carbonyl anions. This is in strong contrast with the results for positive ionization of this target system, where ions attached to (remains of) argon nanosupport are clearly observed. The created anions always desorb from argon.

The data from the two different cluster-beam setups are in excellent agreement with respect to the position (and in most cases also relative intensities) of the bands and Figure 3 and Figure 4 directly demonstrate the power of their combination: while the data from CLUSTER have much better electron-energy resolution and thus reveal sharper structures, the CLUB setup has higher sensitivity and provides yields also for much weaker fragments. The present data represent an important cross-check validation of both experiments.

## Discussion

### Low energy (0–2 eV): DEA suppression upon clustering

As outlined in the Introduction, the DEA to gas-phase iron pentacarbonyl at low energy proceeds via cleavage of one metal–ligand bond

[1]
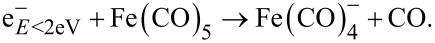


When considering a dimer as the target system, this channel can yield either the Fe(CO)_4_^−^ anion (top panel of Figure 2) or this anion with another monomer unit attached to it (top panel of Figure 3):

[2]
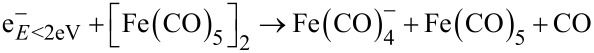


[3]
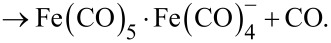


In both cases, the sharp narrow peak close to 0 eV, visible in the gas-phase spectrum (reaction (Equation 1)) disappears. Moreover, when the target dimer is adsorbed on argon nanoparticles with the mean size of 200 atoms, the whole low-energy band is suppressed.

We have evidence [18] that the cross section for the process (Equation 1) is strongly enhanced by the presence of a virtual state in the electron–Fe(CO)_5_ scattering. The origin of such a virtual state is polarizability of Fe(CO)_5_: the incoming electron polarizes the iron pentacarbonyl which leads to their interaction on a long-distance scale. The presence of another species (either another Fe(CO)_5_ molecules or argon) probably leads to electrostatic shielding of such a long-distance interaction: the elementary surrounding decreases the resulting electric field at the target molecule, which reduces its attractive interaction with the incoming electron. This causes the significant cross section drop at very low energies. When such an aggregate is sitting on a much larger argon nanoparticle, the shielding, due to the polarizable nature of argon, may be so effective that the cross section of the whole band at energies *<*2 eV is below the detection limit of the CLUB setup.

Similar shielding has been postulated in electron-irradiation CH_3_I condensed on rare gas films [10–11]. In the vast majority of molecules, the vicinity of a surface usually leads to an enhancement of the DEA cross section when compared to the gas phase. The main reason for this is that the formed transient anion polarizes the surface which leads to the lowering of the anion potential energy curve. However, CH_3_I shows very different behavior: upon condensing on Kr films, its DEA cross section drops significantly when compared to the gas phase (approximately by a factor of 20) [11]. The gas-phase DEA in CH_3_I cross section peaks at 0 eV and the long-range interaction has been shown to strongly enhance its value [19]. The surface environment effectively suppresses these interactions due to its polarizable nature which has an effect of the elimination of this enhancement [9]. It should be noted that the diffuse anion state in CH_3_I is slightly bound which leads to the formation of a vibrational Feshbach resonance, the virtual state scattering can be viewed as the ”slightly unbound” analogue. Nonetheless, the effect of shielding due to polarizability of the surroundings (either surface or clusters) can be expected to be the same.

Massey et al. [20] have recently probed the anion desorption from iron pentacarbonyl thin films condensed on xenon upon electron irradiation and reported no desorption signals below 5 eV. The authors attributed this primarily to low desorption probability of fragments produced at low electron energies. In view of the present results, the shielding of the long-range interactions by the bulk surface could be responsible for such an observation as well.

### Higher electron energy (5–25 eV): self-scavenging

The strong bands at this energy range, seen for all fragment anions, closely resemble in shape the electron energy loss spectra of gas-phase iron pentacarbonyl [14]. This suggests that the production mechanism proceeds via self-scavenging: electronic excitation of one Fe(CO)_5_ molecule, attachment of the slowed-down electron with low residual energy to another cluster constituent and subsequent anion-molecule association reactions:

[4]
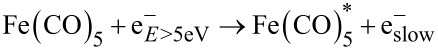


[5]
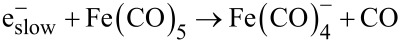


[6]
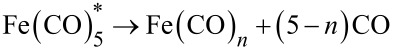


[7]
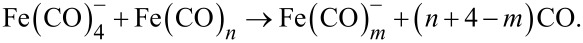


It should be stressed that the proposed mechanism does not need to be viewed as a sequential process: the two involved Fe(CO)_5_ molecules are constituents of the same cluster, the electronic excitation of one of them and attachment of the slow electron to another one can proceed basically simultaneously as a two-center process. This may be important: in view of the low-energy DEA suppression due to screening of the long-range interaction, one would expect process (Equation 5) to be rather ineffective. However, in this situation, the target molecule is not interacting with a free incoming electron, but with an electron that is already interacting with another cluster constituent. This makes the role of long-range forces difficult to asses.

For aggregates on argon nanoparticles, the band between 5 and 10 eV is clearly disappearing with the increasing number of removed ligands. It is thus most probably linked with the number of ligands removed in the step (Equation 6). The origin of the ligand stabilization by argon support cannot be purely mechanistic (caging), because then also other bands would disappear. Interestingly, the disappearing band lies in energy range, where the excitation of the singlet Fe(CO)_5_^*^ states prevails in step (Equation 4), while the band that pertains lies in the energy range for triplet excitations [14,18]. We do not have a good explanation of the origin of such a state-selective ligand stabilization by the argon support.

## Conclusion

We have shown that the iron pentacarbonyl decomposition channel, operative at low electron energy (DEA leading to cleavage of one metal–ligand bond) is gradually suppressed upon the increasing aggregation state of Fe(CO)_5_. When it is in the form of small clusters, the sharp peak at 0 eV disappears. When it is additionally adsorbed on argon nanoparticles with a mean size of 200 atoms, the DEA signal below 2 eV disappears completely. This is probably caused by a suppression of a virtual state due to screening of long-range interactions by the elementary environment, similarly as it was observed for CH_3_I in condensed phase [9,11]. At energies above 5 eV, complex anions are synthesized via self-scavenging, where the processes are described by Equations 4–7. The argon support influences this energy range only very little.

The present findings are well in line with several surface science studies. Hauchard and Rowntree [21] studied electron-induced decarbonylation of Fe(CO)_5_ films on Au(111)/mica using IR spectroscopy. They concluded that massive mixed Fe*_n_*(CO)*_m_* species result from secondary reactions of anion fragments with neighboring Fe(CO)_5_ molecules. The kinetic model revealed two main electron energy ranges for decarbonylation: very low (below ≈2 eV) and intermediate (above 5 eV). This is in very good agreement with the present results on anion synthesis in pure Fe(CO)_5_ clusters. As already mentioned, Massey et al. [20] studied degradation of Fe(CO)_5_ films condensed on a xenon spacer on platinum foil by electron stimulated desorption. They did not observe any anion desorption at electron energies below 4 eV and strong desorption signals in the energy range 5–20 eV. The desorption yields very closely resemble (in shape) the yield from Fe(CO)_5_ aggregates deposited on argon nanoparticles [14], which indicates a dominant role of self-scavenging.

The present findings confirm an important fact: the electron-triggered reactions in a typical FEBID precursor are extremely sensitive to its elementary environment. In the gas phase, the decomposition via anion production proceeds at very low electron energies. Even the presence of several neighboring molecules opens the possibility of a new anion synthesis channel at electron energies above 5 eV (self-scavenging). On the other hand, the presence of a few hundreds of argon atoms suppresses the low-energy channel completely. This low-energy behavior complements our previous results on clusters of FEBID precursors. For example, we have recently shown [13] that the positive ionization, which is very fragmentative in the gas phase, becomes much less destructive in clusters, since the ligands are stabilized by caging. The electronic excitation and subsequent neutral dissociation (very effective in the gas phase [22–23]) manifests itself in clusters via self-scavenging as a synthesis mechanism of mixed metal–ligand species [14]. All in all, the sensitivity to the environment should not be overlooked when attempting to use the results of gas-phase studies for interpretation or optimization of surface and deposition experiments.
